# Identification of Gram negative non-fermentative bacteria: How hard can it be?

**DOI:** 10.1371/journal.pntd.0007729

**Published:** 2019-09-30

**Authors:** Toni Whistler, Ornuma Sangwichian, Possawat Jorakate, Pongpun Sawatwong, Uraiwan Surin, Barameht Piralam, Somsak Thamthitiwat, Chidchanok Promkong, Leonard Peruski

**Affiliations:** 1 Division of Global Health Protection, Centers for Disease Control and Prevention, Atlanta, Georgia, United States of America; 2 Thailand Ministry of Public Health—US Centers for Disease Control and Prevention Collaboration (TUC), Nonthaburi, Thailand; 3 Nakhon Phanom General Hospital, Nakhon Phanom Provincial Health Office, Nakhon Phanom, Thailand; University of Texas Medical Branch, UNITED STATES

## Abstract

**Introduction:**

The prevalence of bacteremia caused by Gram negative non-fermentative (GNNF) bacteria has been increasing globally over the past decade. Many studies have investigated their epidemiology but focus on the common GNNF including *Pseudomonas aeruginosa* and *Acinetobacter baumannii*. Knowledge of the uncommon GNNF bacteremias is very limited. This study explores invasive bloodstream infection GNNF isolates that were initially unidentified after testing with standard microbiological techniques. All isolations were made during laboratory-based surveillance activities in two rural provinces of Thailand between 2006 and 2014.

**Methods:**

A subset of GNNF clinical isolates (204/947), not identified by standard manual biochemical methodologies were run on the BD Phoenix automated identification and susceptibility testing system. If an organism was not identified (12/204) DNA was extracted for whole genome sequencing (WGS) on a MiSeq platform and data analysis performed using 3 web-based platforms: Taxonomer, CGE KmerFinder and One Codex.

**Results:**

The BD Phoenix automated identification system recognized 92% (187/204) of the GNNF isolates, and because of their taxonomic complexity and high phenotypic similarity 37% (69/187) were only identified to the genus level. Five isolates grew too slowly for identification. Antimicrobial sensitivity (AST) data was not obtained for 93/187 (50%) identified isolates either because of their slow growth or their taxa were not in the AST database associated with the instrument. WGS identified the 12 remaining unknowns, four to genus level only.

**Conclusion:**

The GNNF bacteria are of increasing concern in the clinical setting, and our inability to identify these organisms and determine their AST profiles will impede treatment. Databases for automated identification systems and sequencing annotation need to be improved so that opportunistic organisms are better covered.

## Author summary

Infections caused by Gram negative non fermentative bacteria (GNNF), other than the more commonly described *Pseudomonas aeruginosa* and *Acinetobacter baumannii*, constitute an emerging problem. They are not only isolated in nosocomial settings, mainly affecting immunocomprised hosts, but also are opportunistic infections causing invasive disease in rural communities. GNNF bacteria are ubiquitously distributed in the environment and have a high propensity for antimicrobial resistance. Thus, their accurate identification to the species level is important for appropriate patient management. These bacteria are taxonomically heterogeneous, and many isolates are not satisfactorily identified by the standard biochemical assays because of overlapping phenotypic characteristics. In this study we explored using the BD Phoenix automated identification system to improve the identification of GNNF isolates and recognized 92% (187/204) of the previously unidentified isolates; 117 of which were to the species level. Antimicrobial resistance profiles were identified for 50% (94/187) of the isolates identified by the BD Phoenix system. For the remaining 12 unidentified organisms whole genome sequencing was performed. Three web-based programs were used for organism identification and results varied because of differences in the underlying databases used.

## Introduction

The non-fermentative Gram-negative bacteria are widely distributed in the environment and have become increasingly common isolates in the clinical laboratory. Being ubiquitous in nature, they are often disregarded as contaminants. Medically, their pathogenic potential has been proved beyond doubt by their frequent isolation from clinical material and their association with disease. This group of bacteria has emerged as opportunistic pathogens, particularly in immunocompromised hosts, and are difficult to treat because of widespread antibiotic resistance. Due to their taxonomic complexity and phenotypic similarity accurate identification represents a challenge for conventional microbiology.

Automated systems that perform organism identification and antimicrobial susceptibility testing are now the mainstay of clinical microbiology laboratories. The recent implementation of the BD Phoenix and a MiSeq next generation sequencing (NGS) instrument in our laboratory-based surveillance activities has allowed us to re-examine a subset of previously unidentified Gram negative bacilli and determine which genera are contributing to bloodstream infections in rural, agrarian populations in Thailand. An understanding of the uncommon Gram negative non-fermentative (GNNF) bacteria causing invasive disease in these communities should assist in diagnosis and treatment and possibly impact patient outcomes.

## Methods

### Ethical statement

The CDC Human Subjects Review Office determined this protocol to be a routine public health activity not involving human subject research (CGH Determination and Approval number 2014–273).

### Isolate collection

**Fig 1:**
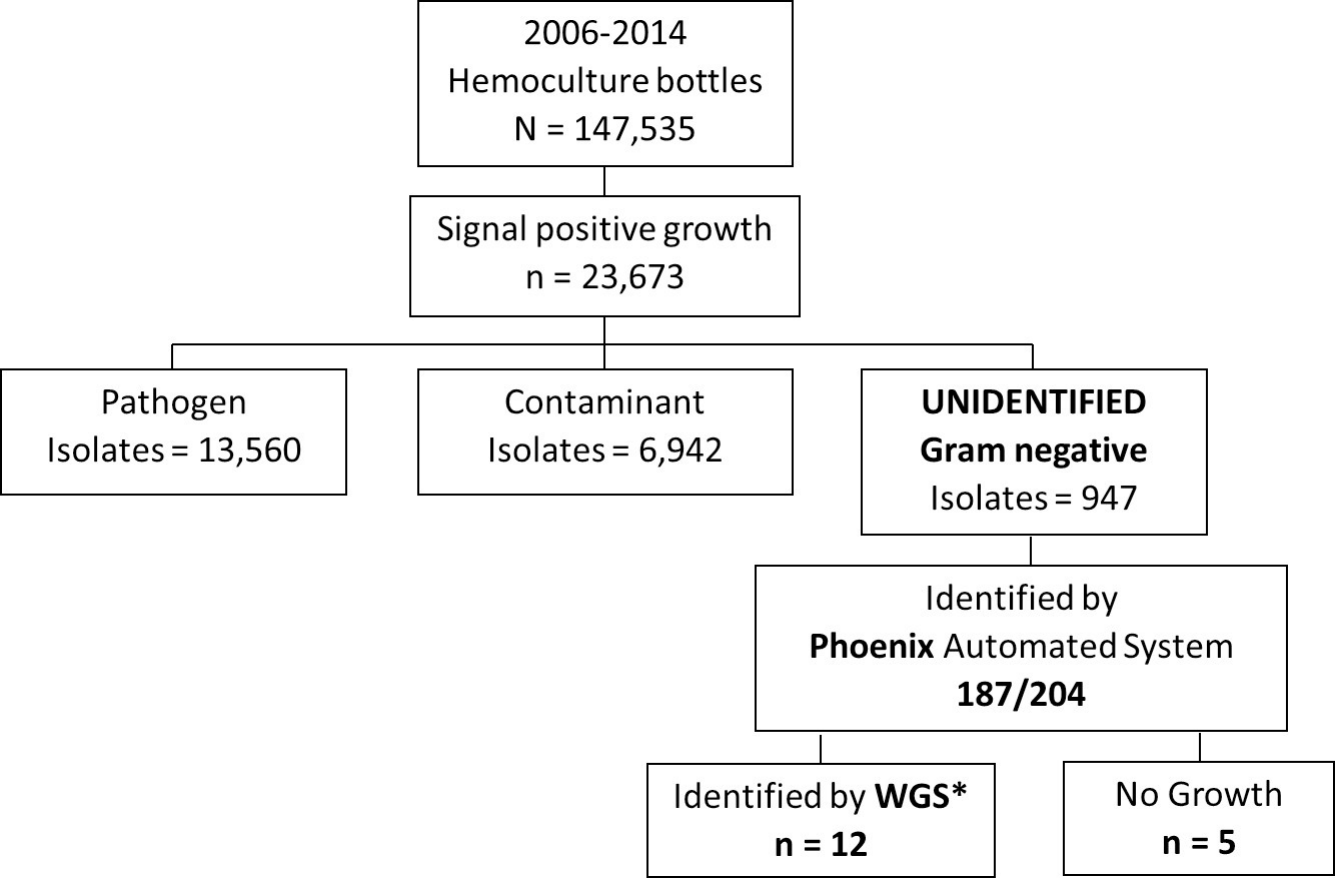
Flow diagram denoting blood culture results from blood-stream infection surveillance in two rural Thai provinces from 2006–2014. *WGS–whole genome sequencing.

Unidentified Gram negative bacterial isolates used in this study (Fig 1) were collected during 2006–2014 as part of a previously published surveillance system [1]. Organisms were taken from the isolate culture collections of the Sa Kaeo and Nakhon Phanom Provincial Health Laboratories that had been previously been characterized using standard biochemical testing methods [2]. A random subset of approximately 20% (n = 204) of the 947 unidentified organisms were used in this study.

### BD Phoenix system procedures

Upon removal from the freezer, isolates were grown on tryptic soy agar with 5% sheep blood. The inoculum for the Phoenix Gram-negative identification and susceptibility combination panel NMIC/ID 55 was prepared according to the manufacturer’s instructions and panels were sealed, logged, and loaded into the instrument for incubation at 35°C (http://legacy.bd.com/ds/technicalCenter/clsi/clsi-Phoenix_GramNegative_V5.15_V4.31.pdf). Kinetic, colorimetric, and fluorescent signals were automatically collected by the instrument every 20 minutes until results were completed. The Phoenix system leads to an identification result when a species or group of species is identified with a ≥90% confidence level [3]. Antimicrobial susceptibility testing (AST) breakpoints were interpreted using the Clinical and Laboratory Standards Institute (CLSI) guidelines from 2016 [4].

### Whole genome sequencing

DNA was extracted from bacterial colonies using the manual Qiagen DNA Mini Kit. Libraries were prepared employing the Illumina Nextera XT DNA Library Prep Kit according to the manufacturer's instructions. Whole genome sequencing was performed on the Illumina MiSeq sequencer (Illumina, San Diego, CA) using Illumina MiSeq Reagent Kit version 2.0 and 300 cycles paired-end runs.

Sequences have been deposited in the NCBI Sequence Read Archive and can be accessed through Biosample identifiers SAMN11784141–52.

### Downstream genome analysis

The whole genome data were analyzed using open access tools through web-based servers that accept raw fastq files. Organism identification for the study isolates were determined using: 1) Center for Genomic Epidemiology (CGE) KmerFinder 3.0 (https://cge.cbs.dtu.dk/services/KmerFinder/) [5] with the selected database bacteria organisms (K: 16, P: ATG) updated July 2018. Run 10/16/2018. This method finds the unique 16-mers (k-mers) in the input sequence and predicts species based on the number of overlapping k-mers between the query genome and genomes in a reference database. The prediction is made at which it has the highest number of 16-mers in common despite of position. 2) Taxonomer (https://www.taxonomer.com/) run in full analysis mode which analyzes the incoming read data in two steps: (a) binning, which classifies reads into top categories, such as Human, Bacteria, Virus, etc. and (b) classification, which further categorizes each read into a particular phylum or species, if sufficiently unique. Bacterial reads are classified against 16S rRNA and bacterial transcriptomes compiled from Greengenes and UniRef50 databases [6]. 3) One Codex (https://onecodex.com/) [7] using the free research account that allows for analysis of 25 samples. One Codex classifies the input files against three databases of microbial genomes and genes a) the RefSeq Complete Genomes, b) the extended One Codex database and c) a targeted loci database.

Results are presented as absolute percentages regardless of phylogeny level so are presented as the percent of total bacterial reads classified.

## Results

**Fig 2:**
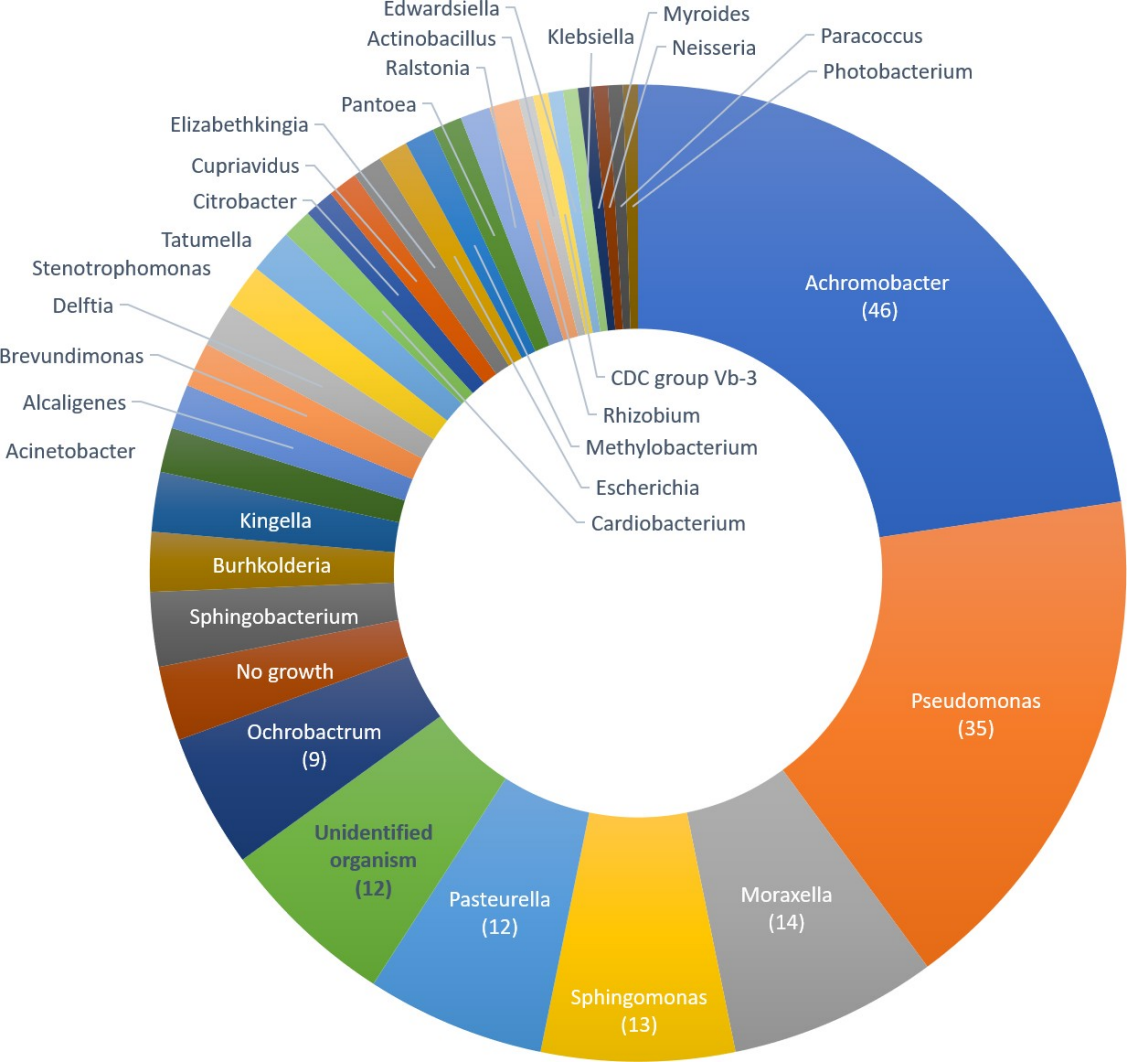
Distribution of previously unidentified isolates at the genus level using BD Phoenix automated identification system.

**Fig 3:**
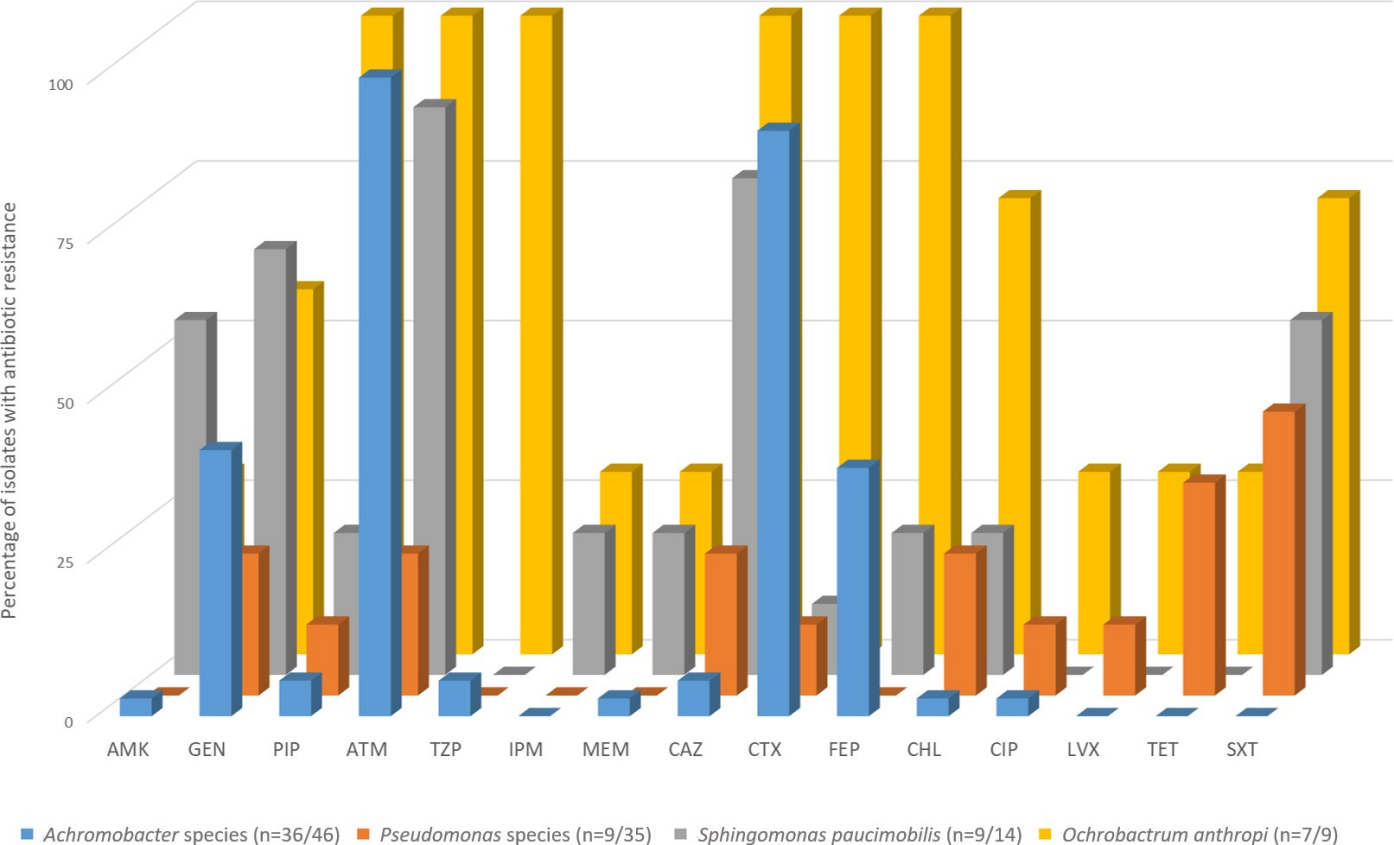
Antibiotic resistance levels by organism using the BD Phoenix identification system. Antibiotic abbreviation by drug class: Aminoglycosides: AMK–Amikacin, GEN—Gentamicin; Beta-lactam: PIP–Piperacillin, ATM–Aztreonam, TZP–Piperacillin-Tazobactam; Carbapenem: IPM–Imipenem, MEM–Meroenem; Cephalosporin: CAZ–Ceftazidime, CTX–Cefotaxime, FEP–Cefepime; CHL–Chloramphenicol; Fluoroquinolone: CIP–Ciprofloxacin, LVX–Levofloxacin; TET- Tetracycline; Folate Antagonist: SXT—Trimethoprim-Sulfamethoxazole. In the figure legend n = number of isolates for which BD Phoenix antimicrobial sensitivity test (AST) data was available/total number of species specific isolates in this study. For several isolates where AST data was not recorded the isolate grew too slowly to meet the growth control criteria cutoff. Clinical and Laboratory Standards Institute (CLSI) 2016 minimum inhibitory concentration break points were applied. Moraxella (n = 14) and Pasteurella (n = 12) species are not included in the BD Phoenix AST database and therefore this data is not available.

Approximately 7% (947/14,507) of all clinical isolates could not be identified by standard biochemical methods. A proportion of these, denoted as “unknown” (204/947 (21.5%); Fig 1), were randomly selected and examined using the BD Phoenix automated identification and susceptibility testing system. Identification to at least the genus level was achieved for 187/204 (91.7%) (Fig 2; S1 Tables). The largest number of isolates identified as *Achromobacter* species (46/204; 22.5%), with nearly 70% of cases being in adults ≥50 years of age (32/46, Table 1). One case appeared to be hospital onset (defined as positive blood cultures obtained >2 days after hospital admission), with blood obtained for blood culture 5 days after admission. All others were community-acquired infections. AST data was available for 36 isolates (78.3%) and all showed resistance to ampicillin, amoxicillin-clavulanate, aztreonam, and cefazolin. Cefepime resistance was determined in 14/36 isolates with the remaining 18 isolates scoring as intermediate (Fig 3; S1 Tables). Ten *Achromobacter* isolates identified by Phoenix had no associated AST data as isolate growth was too slow, preventing the control from reaching the required cutoff value and terminating that portion of the panel. On re-test a similar result was obtained.

**Table 1:** Demographic data associated with the 204 unidentified isolates processed using the BD Phoenix automated identification system, focusing on commonly identified genera.

	GNNF Phoenix ID (n = 204)		*Achromobacter* spp. (n = 46)		*Pseudomonas* spp. (n = 35)		*Moraxella* spp. (n = 14)	
	n	%	n	%	n	%	n	%
**Age Group n**	**188**	**92.2**	**44**	**95.7**	**33**	**94.3**	**13**	**92.9**
(years) 0–4	20	10.6	0	0	7	21.2	1	7.7
5–19	6	3.2	1	2.3	2	6.1	0	0
20–49	42	22.3	11	25.0	4	12.1	3	23.1
50–74	89	47.3	23	52.3	18	54.5	4	30.8
75+	31	16.5	9	20.4	2	6.1	5	38.5
**Sex n**	**73**	**35.8**	**19**	**41.3**	**11**	**31.4**	**4**	**28.6**
Male	37	50.7	8	42.1	6	54.5	3	75.0
Female	36	49.3	11	57.9	5	45.5	1	25.0
**Onset**^a^ **n**	**188**	**92.2**	**41**	**89.1**	**32**	**91.4**	**13**	**92.9**
Community Onset	110	58.5	40	97.6	28	87.5	8	61.5
Hospital Onset	78	41.5	1	2.4	4	12.5	5	38.5
**Outcome**^b^ **n**	**126**	**61.8**	**37**	**84.1**	**22**	**62.6**	**9**	**64.3**
Complete recovery	14	11.1	3	8.1	3	13.6		
Improved	76	60.3	22	59.5	12	54.5	7	77.8
Not improved	23	18.3	7	18.9	6	27.3	1	11.1
Death	13	10.3	5	13.5	1	4.5	1	11.1
**Site n**	**204**	**100**	**46**	**100**	**35**	**100**	**14**	**100**
Nakhon Phanom	140	68.6	41	89.1	20	57.1	11	78.6
Sa Kaeo	64	31.4	5	10.9	15	42.9	3	21.4

^a^ Infection classified as community onset when positive cultures were obtained ≤48 hours after hospital admission; hospital onset after that.

^b^Outcome data was derived from several variables in the surveillance database including “outcome”, “discharge status” and “discharge type”. If the patient was discharged with consent, the classification was “improved”; discharged against advice was recorded as “not improved”.

*Pseudomonas* species were identified in 35/204 isolates (17.2%) of which the most common were *P*. *putida* (11; 31.4%), and *P*. *pseudoalcaligenes* 8 (22.9%). *P*. *oryzihabitans* and *P*. *aeruginosa* had 5 and 4 isolates respectively, and one isolation each of *P*. *luteola*, *P*. *mendocina* and *P*. *stutzeri*. Three *Pseudomonas* isolates were not speciated. The majority of isolates were not able to meet the growth criteria required in the AST control, even upon re-testing, and susceptibility profiles were only available for 9/35 (25.7%) isolates. These were 100% resistant to ampicillin, amoxicillin/clavulanate (augmentin), cefazolin, and cefoxitin, as expected with intrinsic resistance to these antibiotics. All 9 were sensitive to amikacin, cefepime, imipenem and piperacillin/tazobactam (Fig 3; S1 Tables). Twenty percent of cases (7/35) were in children <5 years old (Table 1); and 3/4 likely hospital onset were in children <5 years old.

The third most common isolates were *Moraxella* species (14/204 cases (6.9%)), with 5 being hospital onset infections, and most cases occurring in adults ≥50 years old (9/14; 64.3%). AST profiles were not available as *Moraxella* species are not included in the Phoenix AST database [8].

Other genera identified are *Sphingomonas paucimobilis* (13/204 isolates), twelve isolations of *Pasteurella* species (7 *P*. *multocida*, 2 *P*. *aerogenes*, and 3 *P*. *pneumotropica*), and 9 isolations of *Ochrobactrum anthropi*.

Clinical outcome data was available for 126/204 (61.8%) of case-patients (Table 1), ninety patients (71.4%) had a complete recovery, or their condition was improving at the time of discharge. Twenty-three patients (18.3%) showed no improvement (13 were transferred to another hospital and two discharged against medical advice). Thirteen patients died (10.3%).

AST profiles were not determined for 93/187 (50%) isolates: 44/187 isolates (23.5%) as they were not included in the Phoenix AST taxa (15 different species; S1 Tables). A further 50 (26.7%) isolates grew too slowly so the cutoff value required in the AST growth control was not reached.

Table 2 summarizes the time-to-positivity ((TTP) defined as the time from the start of blood culture bottle incubation to a positive signal) and the patient self-reported antibiotic use within 72 hours of hospital admission. Antibiotic use was extremely high among cases with only 16.9% (31/183) reporting that no antibiotics were used. Blood culture TTP shows that 111/176 (63.1%) of cultures were called within 48 hours (Table 2), despite the high levels of antibiotic usage.

**Table 2:** Summary of patient antibiotic use prior to admission and time-to-positivity (TTP) for blood culture.

		GNNF Phoenix ID (n = 204)		*Achromobacter* spp. (n = 46)		*Pseudomonas* spp. (n = 35)		*Moraxella* spp. (n = 14)	
		n	%	n	%	n	%	n	%
**Antibiotic use within 72h admission** (Self-report)	**n**	**183**	**89.7**	**44**	**95.6**	**31**	**88.6**	**12**	**85.7**
	Yes	133	71.1	26	59.1	23	74.2	10	83.3
	No	31	16.9	12	27.3	7	22.6	2	16.7
	Not sure	19	10.3	6	13.6	1	3.2	0	0
Blood culture TTP*	**n**	**176**	**86.3**	**44**	**95.6**	**31**	**86.1**	**12**	**65.7**
(hours)	≤ 24	25	14.2	1	2.3	4	12.9	3	25.0
	25–48	86	48.9	22	50.0	17	54.8	4	33.3
	> 48	65	36.9	21	47.7	11	35.5	5	41.7

* TTP defined as the time from the start of blood culture bottle incubation to a positive signal.

A total of 17/204 (5.9%) isolates could not be identified by the Phoenix system, five because of no growth. Characterization of the remaining 12 isolates was undertaken through WGS on an Illumina MiSeq platform.

Isolate NA66303 was identified as *Laribacter hongkongensis* by all 3 programs (Table 3; S2 Tables). The One Codex software identified the sample as a low-complexity/isolate and a high confidence identification with 88.8% of reads (n = 960,230) being specific to *L*. *hongkongensis*. CGE KmerFinder mapped 68.4% of Kmers to the strains HLHK9 and LHGZ1. The Taxonomer software classified 977,524/1,081,322 (90.4%) reads as bacterial, with 252,896 (34.1%) being classified to *L*. *hongkongensis* HLHK9.

**Table 3:** Summary of isolate identification using 3 different web-based platforms on whole genome sequencing short read raw data files.

ID	CGE: KmerFinder https://cge.cbs.dtu.dk/services/KmerFinder/	Taxonomer https://www.taxonomer.com/	One Codex https://onecodex.com/
NA45072	*Acinetobacter nosocomialis*	*Wohlfahrtiimonas chitiniclastica*	*Wohlfahrtiimonas chitiniclastica*^*1*^
NA45230	Genus: *Acinetobacter*	Genus: *Acinetobacter*	Genus: *Acinetobacter*
NA45737	*Moraxella osloensis*	Genus: *Enhydrobacter*	Genus: *Enhydrobacter*
NA48754	*Delftia acidovorans*	Genus: *Delftia*	Genus: *Delftia*
NA55273	*Burkholderia cenocepacia*	Genus: *Burkholderia*	*Burkholderia cenocepacia*^*1*^
NA62451	*Roseomonas gilardii*	Family: Acetobacteraceae	*Roseomonas gilardii*^*1*^
NA62784	*Roseomonas gilardii*	Family: Acetobacteraceae	*Roseomonas gilardii*
NA64181	*Pseudomonas oryzihabitans*	Genus: *Pseudomonas*	Genus: *Pseudomonas*
**NA66303**	***Laribacter hongkongensis***	***Laribacter hongkongensis***	***Laribacter hongkongensis***^*1*^
NA69389	Genus: Chryseobacterium*	Genus: *Chryseobacterium*	*Flavobacteriales bacterium*
SA27898	Moraxella osloensis*	*Enhydrobacter aerosaccus*	*Moraxella atlantae*^*1*^
SC13199	*Acinetobacter indicus*	Genus: *Acinetobacter*	*Acinetobacter indicus*

*Low confidence identification: only 1–5% of reads map to the designated organism.

^*1*^One Codex high confidence identification. The sample contains >50% unique genomic content of that organism

The One Codex database was the only program to call isolate SA27898 with high confidence. It classified 81.4% of reads (n = 548,861) of which 79.6% (n = 436,853) were specific to *Moraxella atlantae*, with an estimated depth of coverage at 45X. CGE KmerFinder identified *M*. *osloensis* strain CCUG 350 with a query coverage of 3.1% to this template of 91,679 k-mers. No other significant results were obtained. Taxonomer was able to classify 275,794/548,852 reads (50.2%) as bacterial, but of these only 16,909 (6.1% bacterial or 3.1% of the total number of reads) were assigned to *Enhydrobacter aerosaccus* SK60. A further 8,505 reads were assigned to other *Moraxella* species (S2 Tables). Of note 267,145 reads (48.7%) were unclassified in Taxonomer.

**Fig 4:**
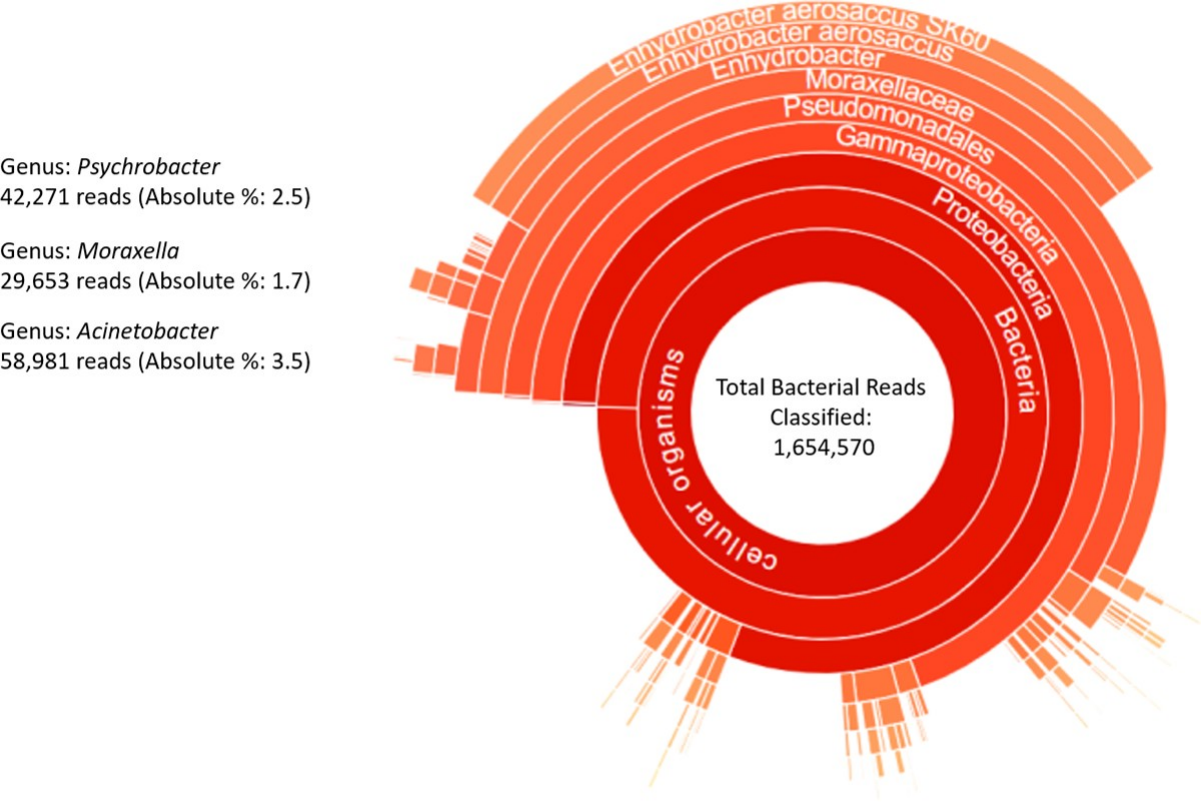
Taxonomer bacterial composition classifier sunburst of isolate NA45737. Reads are classified against 16S sequences and protein sequences from Uniref50. The size of a given sector represents the relative abundance at the read level. Taxonomic ranks are hierarchical with the highest level placed in the center. Reads not classified at the species level, either because they are shared between taxa or represent novel microorganisms, are collapsed to the lowest common ancestor and shown as part of slices that terminate at higher taxonomic ranks (e.g. genus).

NA45737 was identified by both One Codex and CGE KmerFinder as *M*. *osloensis*. In One Codex the isolate was characterized as having no single dominant species and *M*. *osloensis* was a medium abundant species (defined as 5–25% of classified reads) with 13.3% (n = 236,195) of classified reads. Table 4 shows greater detail of the read classifications for NA45737 by phylogeny. At the genus level, even though *Enhydrobacter* has approximately 2.6X more reads than the *Moraxella*, it was classified as the latter because no species was identified to the *Enhydrobacter*. Taxonomer binned 46.6% of reads as bacterial (1,773,485) and a larger proportion as unclassified (2,003,568; (52.7%)), with 29.0% of the bacterial reads assigned to *E*. *aerosaccus* SK60 (Fig 4).

**Table 4:** Details of One Codex analysis of taxonomic grouping for classified reads from isolate NA45737*.

Rank	Taxonomy of classified reads (n = 1,772,214 (46.6%))	
Phylum	Proteobacteria 1,490,804 (84.1%)	
Class	Alphaproteobacteria 655,284 (37.0%)	Gammaproteobacteria 296,499 (16.7%)
Order	Rhodospirillales 655,069 (37.0%)	Pseudomonadales 280,234 (15.8%)
Family	Unclassified Rhodospirillales 654,543 (37.0%)	Moraxellaceae 279,939 (15.8%)
Genus	*Enhydrobacter* 654,553 (37.0%)	*Moraxella* 249,022 (14.0%)
Species	-	*osloensis* 236,195 (13.3%)

*Isolate NA45737 characterized as a mixed/metagenomics sample.

SC13199 was an *Acinetobacter* species isolate as corroborated by all 3 programs. One Codex counted 69.5% (1,386,621/ 1,994,443) classified reads as belonging to the *Acinetobacter* genus. The two highest abundance species were *Acinetobacter* sp. CIP 53.82 (365,836 reads (25.3%)) and *A*. *indicus* (349,113 (24.1%)). Taxonomer classified 61.7% of reads (n = 1,230,514) as bacterial with 15% (190,138/1,230,514) categorize as *Acinetobacter* with no dominant species. CGE KmerFinder identified *A*. *indicus* with 58.8% query coverage. A second isolate, NA45230, was identified as *Acinetobacter* with no speciation. One Codex classified 83.5% of reads of which 97.8% were *Acinetobacter*. Taxonomer mapped 49% of reads and 51.4% were *Acinetobacter* species; CGE KmerFinder had 24.7% of reads mapped to 6 *Acinetobacter* species.

One Codex classified NA69389 as a mixed sample with 2 high abundance species from 69.7% of classified reads: *Flavobacteriales bacterium* (56.8% of classified reads, split between strains UBA896 and UBA3891 (S2 Tables)) and *Chryseobacterium hominis* (18.1%). Taxonomer binned fewer reads as bacterial (56.8%) with 38.5% aligned to the family Flavobacteriaceae and 17.0% associated with the genus *Chrysobacterium*. The dominant species being *C*. *oranimense* (2.2%) and *C*. *gleum* (1.1%). CGE KmerFinder identified *C*. *indologenes* and *C*. *taklimakanense*, each with very low template coverages (3.0% and 1.7% respectively; S2 Tables).

*Wohlfahrtiimonas chitiniclastica* was the dominant species identified in NA45072 by both Taxonomer and One Codex. The former classified 59% of reads as bacterial and 65.1% of those as *W*. *chitiniclastica*. One Codex classified 77.2% of classified reads as *W*. *chitiniclastica*. CGE KmerFinder was unable to identify a dominant organism.

Isolates NA48754, NA55273 and NA64181 showed consensus across the 3 programs at the genus level: *Delftia*, *Burkholderia* (most likely *B*. *cenocepacia*) and *Pseudomonas* respectively (S2 Tables).

The organism associated with isolates NA62451 and NA62784 was clearly identified as *Roseomonas gilardii* by One Codex, with >80% of classified reads linked to *R*. *gilardii* (S2 Tables). In both isolates approximately 16% of reads mapped to *R*. *mucosa*. KmerFinder also identified *R*. *gilardii* as the dominant species with high coverage of chromosome 1 and 2, however Taxonomer was not able to provide a confident identification– 33% of reads were classified to the class Alphaproteobacteria and 10% to the family Acetobacteraceae. Only about 2% or reads were assigned to any particular species.

One Codex was the only program to identify high confidence calls–where the sample had >50% of unique genomic content of that organism (Table 3), and 5/12 (42%) unknowns were such.

## Discussion

The correct and rapid identification of bacteria in a clinical microbiology laboratory, along with antimicrobial sensitivity testing, is an essential step towards the correct treatment of patients. Our analysis of 9 years of blood culture surveillance data shows that traditional diagnostic methodologies resulted in a positive identification in 93.5% of isolates (13,506/14,507). A fair proportion of the unidentified isolates were GNNF bacteria which often present as colorless/pale colonies that lack key metabolic characteristics, which impairs their identification by conventional methods. In 2015, the BD Phoenix automated identification and susceptibility testing system was installed in our laboratory and we retrospectively ran 204 previously unidentified GNNF isolates through the system. This allowed us to identify a further 91.7% (187/204) of isolates to at least the genus level. Due to their taxonomic complexity and high phenotypic similarity, identification to the species level represents a challenge even for the automated systems. In the majority of cases isolates were ubiquitous environmental organisms such as *Achromobacter* and *Sphingomonas* that are likely opportunistic pathogens [9–11]. There are an increasing number of case reports and reviews published suggesting a global increase in achromobacterial disease [10, 12], even so most clinicians remain unclear to their significance (and that of other environmental organisms) when clinically isolated. Additionally, effective treatment can be challenging due to these organism’s intrinsic and acquired multidrug resistance patterns. Our surveillance populations were from rural, agrarian provinces so infections with environmental organisms is not surprising in people who are occupationally exposed. Organisms from over 30 genera were identified. *Moraxella* (14/204) and *Pasteurella* (12/204) species are considered zoonotic pathogens and infection in humans is commonly associated with animal bite, scratch or lick, but infection without epidemiologic evidence of animal contact may occur [13].

Common skin and environmental organisms are often considered likely contaminants [14], however it is difficult to decide if their presence represents a clinically important infection or a false-positive result of no clinical consequence. In our study the contamination rate was around 4.7% (Table 1) and throughout our study efforts were made to reduce contamination through on-going training for nurses on percutaneous blood collection, use of chlorhexidine and alcohol for sterilization of the venipuncture site in adults [15] and tincture of iodine in children [16] using appropriate contact times, disinfection of culture bottle tops and single needle use for bottle inoculation. TTP has been suggested as a marker of bacterial load and used to distinguish bacteremia from contamination. Some studies suggest that cultures positive 3 to 5 days after incubation are more likely to represent contaminants [17, 18]. In our study 63% of cultures were positive within 48 hours and 81.3% (143/176) within 72 hours; and several other factors should be considered i.e. blood volume inoculated and antibiotic presence which make TTP difficult to interpret. It is critical to recognize that these isolates may represent true bacteremias and if untreated due to misinterpretation as contaminants could result in devastating consequences. Isolations of GNNF bacteria have long been disregarded as probable contaminants, but have recently emerged as important healthcare associated pathogens [19]. Our data however, suggests that few of these were nosocomial infections, but likely community-acquired, with many occurring in adults > 50 years old possibly associated with weakened immune systems. Lack of data on the disease epidemiology is a great obstacle to improve patient quality of care, which is further compounded by the lack of antimicrobial resistance data. For example both *Moraxella* and *Pasteurella* species are not included in the Phoenix AST database [8], either because of the low probability of occurrence or special growth requirements. With the significant increase in the incidence of multidrug-resistant pathogens in recent years, the high resistance rates seen in several of the GNNF organisms is concerning.

Traditional diagnostic technologies are insufficient for the identification of organisms not usually considered pathogenic. This limited scope has created bias in what we know about infection and the microbes capable of causing human disease. Our study illustrates that a small, but significant portion of ubiquitous environmental bacterial species can be true pathogens. In our laboratory, these limitations were overcome through the use of an automated microbial identification system, the BD Phoenix.

There was an even smaller proportion of isolates not identified through this system, and we attempted to use WGS to fill this gap. This is possible as small, affordable instrumentation such as the Illumina iSeq or MiSeq, along with decreasing sequencing costs, make WGS an attractive option for lower throughput clinical microbiology and public health laboratories. This does not allow for comprehensive detection of pathogens from clinical samples (metagenomics analysis) but expands on conventional diagnostic testing where it fails to detect the etiologic agent. Targeted 16S rRNA NGS kits are recently available for specimen microbiome composition profiling and offer a cost-effective method for bacterial taxonomic classification, similarly viral genera can be targeted by combining VirCapSeq-VERT and unbiased NGS workflows [20, 21].

Our study illustrates the value of isolate WGS: 12 previously unidentified isolates by conventional or automated methods were classified at least to the genus level. Of note, WGS also allows for examination of antimicrobial resistance genotypes, but this was beyond the scope of this study. The biggest challenge we faced with the introduction of NGS to our laboratory was the data analyses. Laboratory scientists generally lack experience in NGS short-read sequence bioinformatics and in low- or middle- income countries, laboratory computing resources are often limited and unable to handle large data sets. Fortunately, there are rapid, user-friendly web-based tools that can be applied without large investments in trained personnel or computational infrastructure. We chose three services accessible through personal computers with no requirements for computational infrastructure on the user side. For both the Phoenix automated identification system and the NGS web-based tools, the bacterial identification is highly dependent on the reference databases used. As long as there are similar microbes in the database unknowns can be identified. We believe the differences in performance between the selected platforms is a function of the reference database used. For bacterial identification Taxonomer uses 16S rRNA gene sequences from the Greengenes database and the bacterial subset of UniRef50 [6]. The KmerFinder database is a monthly updated extraction from the National Center for Biotechnology Information of whole bacterial genomes, and only contains genomes that have registered taxonomy associated [22]. One Codex has a database collected from public and private sources that is curated through manual and automated steps to remove low quality or mislabeled records [7]. Sequence reference databases are heavily biased toward common pathogens at the expense of environmental microbes and commensals. These biases, sequence inaccuracies and incompleteness are challenges for moving the field forward. Databases should include accurate annotations and high-quality reference sequences that provide a true diversity of strains. Kirstahler et al. [23] found that 43% of bacterial reference genomes, particularly incomplete ones, contained ambiguous sequences and removing these from databases reduced the number of false positive hits. This supports the need for curated microbial genome databases. Our results illustrate that reads derived from taxa that are absent from databases can result in false-negative and false-positive classifications, especially at the genus and species level. Different measures are provided in the outputs from the three programs and no parameters have been clearly defined to qualify an identification. Consistent application of identification parameters would help move this approach forward.

The advantages of web-based analysis for laboratories is obvious. The intensive computing happens on a server located anywhere in the world, which reduces the necessity to invest in expensive computers and bioinformatics resources, and lets scientists interact with their data immediately and directly. Results are obtained rapidly and can be presented in a user-friendly interface such as a sunburst chart (Fig 4) with a dynamic graphic view that presents corresponding species’ proportion. The choice of an appropriate analytical tool is crucial and not trivial. Results still require interpretation by a microbiologist, and an understanding of the analytical limitations is essential, as is clearly evident in our data. Even with these limitations NGS technology should be considered an essential supplement to culture-based methods, particularly where standard diagnostic tests consistently fail to identify the causative pathogen. The development of targeted sequencing kits will enable organism identification from specimens on lower throughput instruments such as the iSeq and MiSeq, a crucial step in any clinical/public health microbiology investigation.

The commonest GNNF bacteria (*Acinetobacter* spp. and *P*. *aeruginosa*) are widely considered common nosocomial infections [24–26], and this was evident in the two Thai rural provinces under surveillance here; Rhodes et al. [1] showed these were the second and sixth commonest hospital onset infections respectively. In this same study they were the 7^th^ and 10^th^ commonest pathogens causing community onset bloodstream infections. Our data also illustrates that the less commonly identified GNNF isolates are predominantly community onset (Table 1). This is a concern as the phenotypic similarity and taxonomic complexity makes these organisms frequently difficult to identify, and their clinical significance may be difficult to determine as these organisms rarely cause invasive infections. From the literature we discover that *L*. *hongkongensis* causes non-bloody acute diarrhea with cases linked to eating freshwater fish [27]. It can cause invasive [28, 29] and even fatal disease [30], and has mainly been described in East Asia [28, 31] but a worldwide distribution is suggested by case reports from Europe [32] and North America [33]. The few reported cases of *W*. *chitiniclastica* infection globally show septicemia and skin and soft tissue infections [34–36]. Numerous cases are documented in relation to maggot infestations and several affected were homeless [34, 37, 38]. Bacteremic cases have been reported from North and South America [34, 38], Europe [37], the United Kingdom [39]. As with our unidentified isolates, *Roseomonas* species are considered opportunistic pathogens because of their low pathogenic potential in humans, and *R*. *gilardii* is most frequently related to human infections [40–42]. It is significantly associated with septicemia and underlying immunocompromised conditions [40, 43]. Several cases have been presented of catheter-related bacteremia with immunosuppression raising [41, 43] the possibility that *Roseomonas* species may be a part of the normal skin flora of humans. Finally *M*. *atlantae* infections are rarely reported in the literature and the few cases of bacteremia appear to all have underlying conditions predisposing them to infection [44, 45]. These organisms are rare, difficult to identify and are easily overlooked so it is likely that their occurrence is underestimated. The clinical significance and appropriate therapy for patients with these bacteremias are not well studied. This together with the emerging challenge of multi-drug resistance, is of serious concern for treatment.

The use of WGS in public health or clinical microbiology laboratories would provide unparalleled improvements in pathogen identification, antibiotic resistance detection, and outbreak investigations, however, this capacity is severely hindered by lack of common standards. Regulatory agencies have not yet provided (or even proposed) standard guidelines, testing has not been standardized, and benchmarks have not been set. External quality assurance and proficiency testing programs are in progress. Analytical pipelines with well-curated, continually updated reference database are also important components to implementation. Once these hurdles have been achieved the incorporation of NGS into clinical and public health routine workflows is achievable.

## Disclaimers

Findings and conclusions presented in this paper represent the views of the authors and do not necessarily official position of the U.S. Centers for Disease Control and Prevention or the institutions with which the authors are affiliated.

## Supporting information

S1 Tables BD Phoenix summary data. Summary data extracted from the BD Phoenix automated identification system reports for each isolate run on the combination panel NMIC/ID 55.
(XLSX)

S2 Tables WGS analysis summaries. Collated data from results generated by the 3 web-based platforms: KmerFinder, Taxonomer and One Codex for the whole genome sequence data from 12 unidentified isolates.
(XLSX)
